# The Activity of Phytotherapic Extracts Combined in a Unique Formulation Alleviates Oxidative Stress and Protects Mitochondria Against Atorvastatin-Induced Cardiomyopathy

**DOI:** 10.3390/ijms26104917

**Published:** 2025-05-20

**Authors:** Maria Gemma Nasoni, Serena Benedetti, Erik Bargagni, Sabrina Burattini, Riham Osman, Michela Battistelli, Francesca Luchetti

**Affiliations:** Department of Biomolecular Sciences, University of Urbino Carlo Bo, 61029 Urbino, Italy; serena.benedetti@uniurb.it (S.B.); e.bargagni@campus.uniurb.it (E.B.); sabrina.burattini@uniurb.it (S.B.); francesca.luchetti@uniurb.it (F.L.)

**Keywords:** cardiomyocyte, statins, myopathy, mitochondria, oxidative stress, natural products

## Abstract

Statins, in addition to their main beneficial lipid-lowering effects (lowering cholesterol and LDL levels), have many additional adverse effects. Among them, the most common is skeletal myopathy. Mitochondria not only play a pivotal role in statin-induced adverse skeletal muscle effects but also seem to be involved in the adverse effects of statins on human cardiac function. However, given that similar oxidative phosphorylation pathways are relevant in skeletal and cardiac muscles, whether long-term statin treatment may alter cardiac muscle is currently unknown. Natural products have been widely employed in skeletal muscle disorders thanks to their antioxidant and anti-inflammatory properties. The purpose of this study was to evaluate the effects of a novel phytotherapic formulation (PF) composed of *Curcuma* and *Boswellia* essential oils, *Harpagophytum procumbens* root, and *Bromelain* on the human AC16 cell line in an in vitro model of atorvastatin-induced cardiomyopathy. Our results showed that atorvastatin decreased cell viability by approximately 50% and induced ROS production and mitochondrial structural damage. Interestingly, supplementation of cells with PF reduced oxidative stress by 20%, improved mitochondrial reshape and function, and restored the expression of the Nrf2/HO-1/GPX4 axis. These results provide new insights into statin-induced cardiomyopathy and suggest the employment of PF as a promising agent in the recovery of cardiac function.

## 1. Introduction

Statins (3-hydroxy-3-methyl-glutaryl coenzyme A reductase inhibitors, HMG-CoA reductase inhibitors) impair cholesterol production by inhibiting the synthesis of mevalonate, a critical intermediary product in the cholesterol pathway [1]. Statins are among the most effective treatments for preventing cardiovascular diseases by decreasing plasma triglycerides and inducing a modest increase in high-density lipoprotein (HDL) cholesterol. In addition to their cholesterol-lowering effect, statins are known to contribute to the stabilization of atherosclerotic plaques, improvement of endothelial function, and show anti-inflammatory, antioxidant, and anti-thrombotic effects [2,3]. Although statins are generally considered well tolerated, their wide use has shed light on adverse effects. The most common side effect is myotoxicity [4]. An estimated 15% of statin users experience muscle side effects, including myalgia and rhabdomyolysis [5]. There are several mechanisms that are proposed to be involved in statin-induced myopathy. Statins cause inhibition of HMG-CoA reductase, resulting in the lowering of several other intermediates of the pathway. The effect of statins on the mevalonate pathway reduces CoQ10 levels, inhibits mitochondrial electron transport chain (ETC) complexes by increasing ROS production, disrupts mitochondrial membrane potential, decreases mitochondrial DNA (mtDNA) copy number, interferes with mitochondrial swelling, and releases cytochrome c [6,7]. Because skeletal muscle is an energy consumer and strictly depends on mitochondrial activity, mitochondrial dysfunction plays a pivotal role in the pathogenesis of statin-induced myopathy, which impairs muscle function and affects muscle morphology. Several studies conducted in both in vitro and in vivo models indicate that oxidative stress is the principal cause of the toxicity associated with statins [8,9].

Mthembu and coworkers demonstrated that prolonged use of statins may impair mitochondrial function [10]. Specifically, statins can inhibit the activity of mitochondrial complex III, a crucial component in the electron transport chain, resulting in the generation of ROS. Interestingly, it has been demonstrated that high doses of statins increase oxidative stress by reducing the glutathione (GSH) content and promoting the formation of oxidized lipids in the brain and plasma of mice [8].

Negative effects of statins on the mitochondrial function of cardiomyocytes have, however, been recently described in vitro and in vivo. In cardiomyocytes, upon long-term (i.e., 7 weeks) exposure in mice, inhibition of Akt/mTOR signaling altered cardiac and mitochondrial ultrastructure [11]. Furthermore, it has been demonstrated that statins significantly enhanced cardiac troponin release after moderate exercise in humans [12]. Among different statins, atorvastatin is a lipophilic statin with greater binding affinity to HMG-CoA reductase. Atorvastatin has been reported to induce ferroptosis by reducing antioxidant cellular defenses and increasing lipid peroxidation and mitochondrial dysfunction, leading to muscular cell death [13].

While the adverse effects of statins on skeletal muscle have received much attention, remarkably little is known about the effects on the heart, our body’s hardest working muscle.

It is well established that oxidative stress plays a significant role in the development of cardiomyopathy and represents one of the most important causes of cardiomyocyte apoptosis [14]. Oxidative stress can damage cardiomyocytes through various mechanisms, including lipid peroxidation, protein oxidation, and DNA damage. Mitochondria are particularly vulnerable to oxidative stress, and their dysfunction can worsen the condition. Accumulating evidence indicates that sirtuins play a key role in oxidative stress. A study on cardiac-specific SIRT1 knockout mice demonstrated that SIRT1 could promote the expression of mitochondrion-related genes, such as nuclear respiratory factor 2 (Nrf2) and mitochondrial transcription factor A (Tfam), to enhance cardiomyopathy via PGC-1α deacetylation. Additionally, the SIRT1/PGC-1α pathway is also involved in the inhibition of mitochondrial fission and apoptosis in diabetic hearts [15].

Natural products have been attractive sources for centuries to counteract various disease conditions due to their great structural and functional diversities and limited adverse effects in human beings. To date, some natural compounds, namely, terpenoids, polyphenols (resveratrol, curcumin), flavonoids (quercetin, apigenin), vitamin D, etc., are widely known to promote muscle strength, mitochondrial biogenesis, and to reduce hydrogen peroxide production as well as inflammation in skeletal muscle [16]. Specifically, quercetin has been shown to reduce the production of ROS/RNS and the Bcl-2-associated X (Bax)/Bcl-2 ratio, suppressing apoptosis in the C2C12 cell line. Additionally, resveratrol was demonstrated to produce beneficial cardiac effects by enhancing the activation of SIRT1 [17]. Ursolic acid, a pentacyclic lipophilic triterpenoid, was reported to enhance the amount of brown fat in skeletal muscle via increasing the expression of uncoupling protein 1 (UCP1) and the GSH content in mitochondria, contributing to mitochondrial biogenesis and reducing oxidative stress [16]. However, evidence of natural compounds applied in the treatment of myocarditis has not been fully established.

Since the potential harmful cardiac effects of statins that have emerged in several in vitro and in vivo animal studies have not been observed clinically, there is a need to better understand the underlying mechanisms, especially those that protect the heart [18]. The aim of this study was to investigate the protective effects of a novel natural phytotherapic formulation (PF), which includes the essential oils of *Curcuma* and *Boswellia*, *Harpagophytum procumbens* root, and *Bromelain*, on a model of atorvastatin-induced cardiac injury in the human AC16 cell line.

## 2. Results

### 2.1. Scavenging Capacity of Phytotherapic Compounds by DPPH Assay

**Figure 1:**
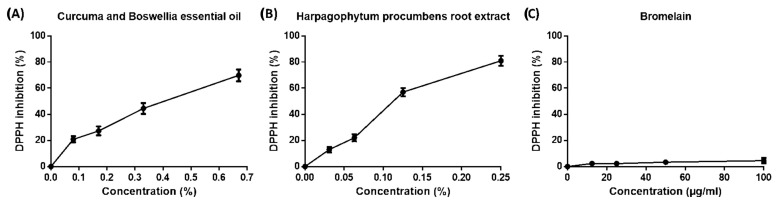
**Scavenger activity of the phytotherapic compounds against DPPH radicals.** The DPPH assay was used to investigate the antioxidant capacity of (**A**) *Curcuma* and *Boswellia* essential oils, (**B**) *Harpagophytum procumbens* root extract, and (**C**) *Bromelain*. Data are expressed as DPPH inhibition (%) ± SD (*n* = 3).

The ability of the phytotherapic extracts to act as antioxidants was evaluated using the DPPH radical scavenging assay. The DPPH assay revealed that the essential oils of *Curcuma* and *Boswellia* and the extract of *Harpagophytum procumbens* root showed significant scavenger effects against the DPPH radical with EC_50_ values equal to 0.4 ± 0.03% and 0.13 ± 0.02%, respectively (Figure 1A,B). On the contrary, *Bromelain* did not show appreciable radical scavenging activity within the range of concentrations tested (EC_50_ > 100 µg/mL) (Figure 1C).

### 2.2. Assessment of the Cellular Compatibility of the Phytotherapic Formulation (PF): Dose–Response Study in AC16 Cell Line

**Figure 2:**
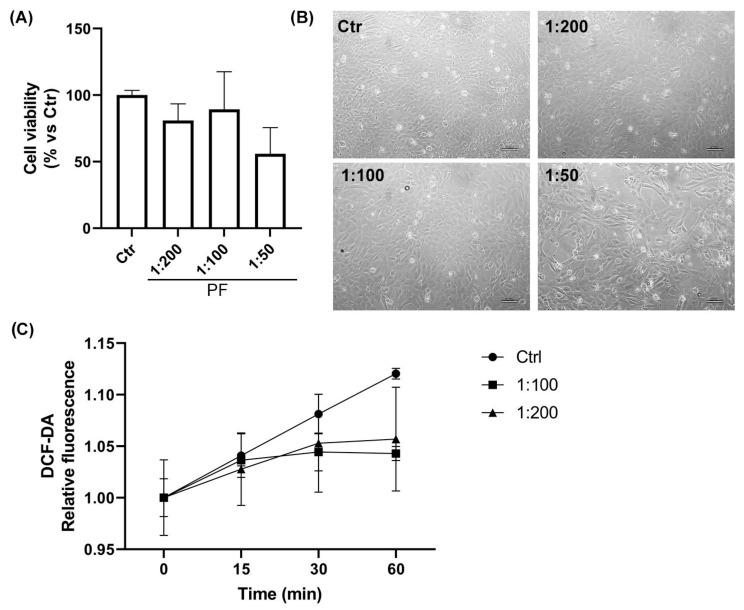
**Evaluation of cell viability and PF antioxidant properties in AC16 cells.** (**A**) Trypan Blue was employed to monitor human AC16 cell viability after 24 h treatment at different dilutions of PF. (**B**) Bright field images obtained by optical microscopy after 24 h of PF treatment. Scale bar: 100 μm. (**C**) Intracellular oxidation levels during 1 h of incubation with PF in AC16 cells. Data collected are presented as mean ± SD (*n* = 3).

In order to identify a compatible and efficacious dose of the phytotherapic formulation (PF) for the AC16 cell line, the cell viability and basal antioxidant activity after PF administration were analyzed using the Trypan Blue and DCFH-DA assays, respectively. As reported in Figure 2A, no significant cytotoxic effects were observed on cell viability with the tested serial dilutions of PF (1:200 and 1:100) after 24 h of treatment. However, the highest concentration of PF (1:50) showed a reduction in cell viability by approximately 40%, as also confirmed in the optical microscopy images (Figure 2A,B). Regarding the antioxidant properties of PF, the lowest concentrations tested (1:100 and 1:200) confirmed the scavenger activity of the phytotherapic extracts. Figure 2C shows a basal reduction in intracellular ROS levels compared to the control condition during 1 h of PF supplementation. On the basis of these results, PF diluted 1:100 was the concentration chosen for the following experiments.

### 2.3. Atorvastatin Induces AC16 Cell Damage in a Dose-Dependent Manner

**Figure 3:**
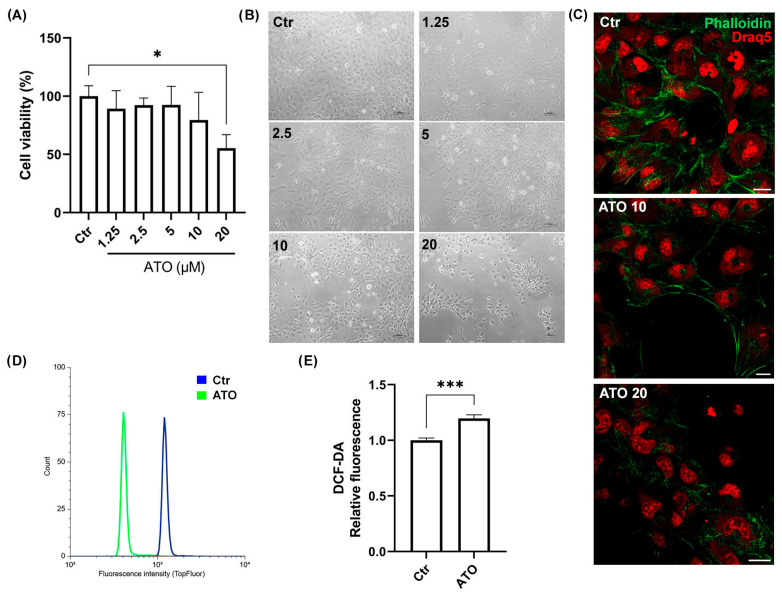
**Dose–response study of ATO in AC16 cell line.** (**A**) Trypan Blue was used to evaluate human AC16 cell viability after 24 h treatment at increasing concentrations of ATO. * *p* < 0.05 vs. control. Data are expressed as mean ± SD (*n* = 3). (**B**) Bright field images of AC16 cells treated for 24 h with increasing concentrations of ATO. Scale bar: 100 μm. (**C**) Representative images of immunofluorescent staining for F-actin (green) with nuclear staining (red) in various groups (magnification, 63×. Scale bar: 20 μm). (**D**) Representative flow cytometry histograms of Bodipy-TopFluor cholesterol fluorescence in control cells (blue line) and cells treated with 20 μM ATO (green line). (**E**) The histogram shows the increment of intracellular ROS after 1 h of incubation with 20 μM ATO (*** *p* < 0.001 vs. control). Data collected are presented as mean ± SD (*n* = 3).

In order to evaluate the myotoxicity of atorvastatin (ATO) in the AC16 cell line, we initially performed cell viability tests after 24 h with increasing concentrations of ATO (1.25, 2.5, 5, 10, and 20 μM). The results reported in Figure 3A show a dose-dependent reduction in viable cells, but only the highest concentration significantly suppressed AC16 cell viability by approximately 40% compared to the control condition (* *p* < 0.05). In Figure 3B, AC16 bright field images obtained with an inverted microscope are shown. Increasing doses of ATO caused progressive abnormal changes, such as round cell shape, cell shrinkage, and reduced density. Fluorescent microscopy analysis with Phalloidin labeling of the actin cytoskeleton also confirmed morphological structure alterations. In Figure 3C, it is possible to observe that, in AC16 control cells, F-actin was distributed homogeneously, surrounding the cell and forming a complete and continuous actin band. On the other hand, cell treatment with the highest concentrations of ATO (10 and 20 μM) for 24 h led to a disordered distribution of F-actin with complete loss of the F-actin cytoskeleton structure in cells treated with 20 μM. In addition, cytofluorimetric analysis of total intracellular cholesterol confirmed a marked reduction in intracellular cholesterol with 20 μM ATO compared to control conditions (Figure 3D). Moreover, the DCFH-DA fluorescence assay revealed that 20 μM ATO administration to AC16 cells led to the early induction of ROS, as documented by the significant increment of intracellular oxidation levels after 1 h treatment (*** *p* < 0.001) (Figure 3E). Therefore, 20 μM ATO was the concentration chosen for the following experiments.

### 2.4. Effect of the Phytotherapic Formulation on Cell Viability and ROS Production in ATO-Treated Cells

**Figure 4:**
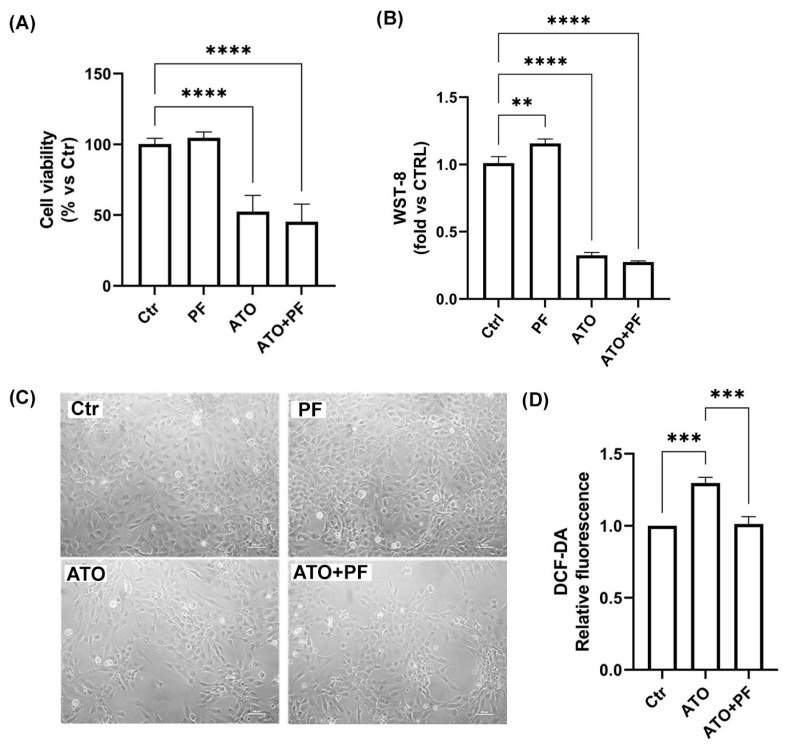
**Evaluation of cell viability and PF antioxidant properties in ATO-treated cells.** (**A**) Trypan Blue was employed to assess AC16 cell viability after ATO treatment following recovery with or without PF. **** *p* < 0.0001 vs. control. Data collected are presented as mean ± SD (*n* = 3). (**B**) CCK-8 assay to evaluate AC16 cell proliferation after ATO treatment following recovery with or without PF. ** *p* < 0.01, **** *p* < 0.0001 vs. control. Data collected are presented as mean ± SD (*n* = 3) (**C**) Representative bright field images of AC16 cells obtained using inverted microscopy for each condition. Scale bar: 100 μm. (**D**) Intracellular oxidation levels after 1 h of ATO incubation with or without PF administration in AC16 cells. *** *p* < 0.001 vs. control; *** *p* < 0.001 vs. ATO. Data collected are presented as mean ± SD (*n* = 3).

The first goal of our study was to determine the effect of PF on cell death and the oxidative state against ATO-induced damage in AC16 cells. Cell viability tests were performed after 24 h of 20 μM ATO treatment and an additional 24 h with or without PF supplementation (1:100). The results reported in Figure 4A confirmed the reduction in viable cells with ATO treatment to approximately 50% compared to the control condition (**** *p* < 0.0001). However, recovery with PF seemed to not influence cell viability (**** *p* < 0.0001 vs. Ctr). The CCK-8 assay also confirmed the data showing a significant reduction in the presence of ATO and also in cells recovered with PF (Figure 4B). In Figure 4C, representative bright field images of AC16 cells obtained with the inverted microscope are shown. Next, we evaluated intracellular ROS production by monitoring changes in DCF-DA fluorescence to demonstrate whether PF attenuated ATO-induced oxidative stress in AC16 cells (Figure 4D). Cells treated with 20 μM ATO showed a remarkable increase in DCF-DA fluorescence intensity compared to control cells. Interestingly, recovery with PF significantly reduced ROS generation (*** *p* < 0.001).

### 2.5. PF Preserves Mitochondrial Ultrastructure in ATO-Treated Cells

**Figure 5:**
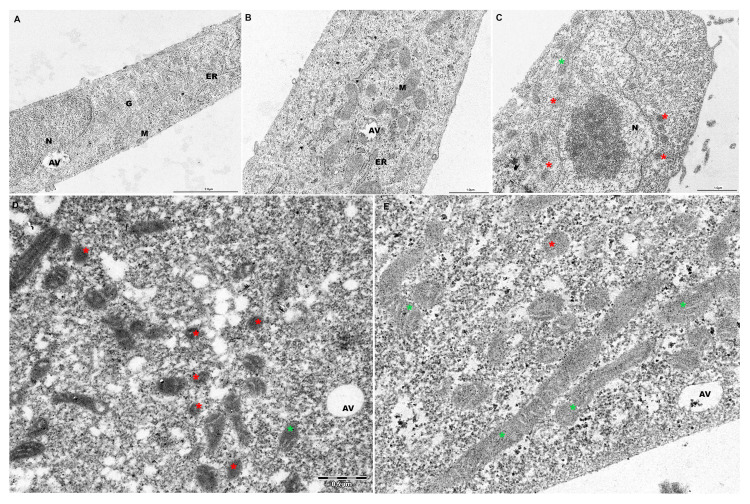
**Transmission electron microscopy images of mitochondrial ultrastructure in AC16 cells.** The ultrastructure images of a control cell (**A**) and PF-treated cell (**B**) show regular cellular morphology and healthy mitochondria in AC16 cells. ATO-treated cells presented altered morphology and numerous shrunk mitochondria with increased membrane density ((**C**), red asterisks). PF supplementation reduced ATO-induced mitochondrial damage, with increased tubular organization and elongated mitochondria with straight and parallel cristae ((**D**,**E**) green asterisks). AV, autophagic vacuole; G, Golgi apparatus; M, mitochondria; ER, endoplasmic reticulum; N, nucleus. Scale bars: 1 and 2 μm.

Transmission electron microscopy (TEM) was performed to investigate morphological features and mitochondria changes after ATO and PF treatments. As shown in Figure 5, AC16 control and PF-treated cells exhibited regular cell membrane structures and the presence of well-preserved cytoplasmic organelles (Figure 5A,B). In particular, numerous tubular mitochondria with parallel cristae appeared after PF supplementation (Figure 5B). Evident morphologic alterations in cell membrane structure and numerous shrunk mitochondria with increased membrane density were observed in ATO-treated cells (Figure 5C, red asterisks). PF treatment remarkably reduced ATO-induced mitochondrial damage, increasing mitochondrial length and tubular organization with regular cristae (Figure 5D,E, green asterisks).

### 2.6. PF Reverses Mitochondrial Dysfunction Induced by Atorvastatin by Improving Mitochondrial Reshaping

**Figure 6:**
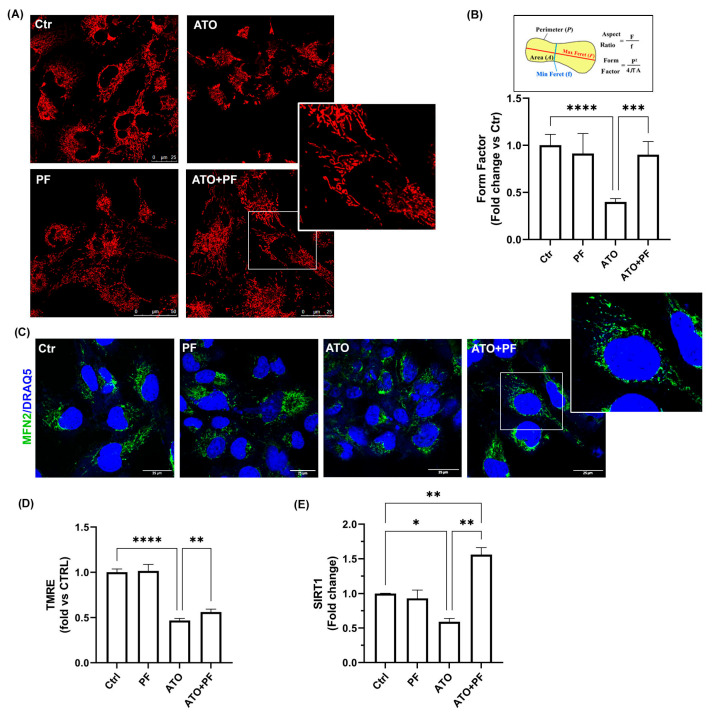
**Effect of PF on mitochondrial network in ATO-treated cells.** (**A**) Representative confocal images of mitochondrial morphology in Ctrl, PF, ATO, and ATO+PF cells. Cells were stained with 200 nM MitoTracker Deep Red (MTDR). Scale bars: 25/50 μm. (**B**) Schematic representation of Form Factor (FF) morphological meaning and FF quantification in Ctrl, PF, ATO, and ATO+PF cells. **** *p* < 0.0001 vs. control cells; *** *p* < 0.001 vs. ATO cells. Data are expressed as mean ± SD (*n* = 3). (**C**) Confocal images of intracellular localization of MFN2 in Ctrl, PF, ATO, and ATO+PF cells. Scale bar: 25 µm. (**D**) TMRE fluorescence quantification in Ctrl, PF, ATO, and ATO+PF cells. **** *p* < 0.0001 vs. control cells; ** *p* < 0.01 vs. ATO cells. Data are expressed as mean ± SD (*n* = 3). (**E**) Relative quantification of SIRT1 mRNA expression levels after 24 h treatment with ATO with or without PF supplementation. Data are expressed as mean ± SD (*n* = 3). * *p* < 0.05, ** *p* < 0.01 vs. control cells; ** *p* < 0.01 vs. ATO cells.

Morphological changes observed by electron microscopy were analyzed via confocal microscopy by measuring the Form Factor (FF) parameter (Figure 6A,B). Mitochondrial staining revealed damaged and fragmented mitochondria in ATO-treated cells, with a significant reduction in FF compared to the control condition (Figure 6A,B; **** *p* < 0.0001). PF supplementation was able to improve the FF parameter, indicating a better-preserved and elongated mitochondrial morphology (Figure 6A,B; *** *p* < 0.001 vs. ATO). We also examined mitochondrial fusion-related factor MFN2 protein by confocal immunofluorescence. The images confirmed that PF-supplemented cells had a higher level of labeling compared to ATO-treated cells, as revealed by the cytoplasmic punctate labeling of MFN2 protein (Figure 6C, enlarged insert), suggesting that PF administration was able to improve the mitochondrial dynamic process. In order to investigate mitochondrial function, the mitochondrial membrane potential (MMP) was assessed. TMRE staining revealed that MMP dramatically decreased after ATO treatment, while PF supplementation affected MMP, increasing its levels (Figure 6D, **** *p* < 0.0001 vs. Ctr; ** *p* < 0.01 vs. ATO). Furthermore, we analyzed SIRT1 expression in order to investigate mitochondrial biogenesis. The results showed that ATO significantly decreased SIRT1 expression and recovery with PF significantly induced a marked increase in its expression (Figure 6E, ** *p* < 0.01 vs. Ctrl and ATO).

### 2.7. PF Restores Nrf2/HO-1/GPX4 Pathway Involved in Myotoxicity Induced by ATO

**Figure 7:**
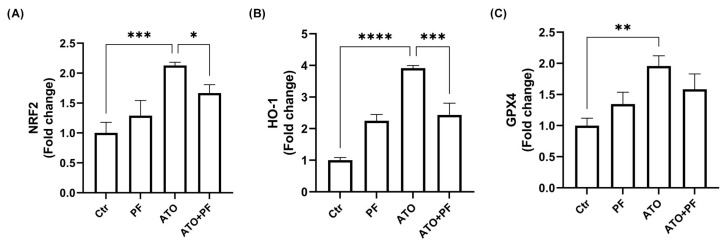
**PF restores Nrf2, HO-1, and GPX4 expression induced by ATO.** Relative quantification of Nrf2 (**A**), HO-1 (**B**) and GPX4 (**C**) mRNA expression levels after 24 h treatment with ATO with or without PF supplementation. Data are expressed as mean ± SD (*n* = 3). ** *p* < 0.01, *** *p* < 0.001, **** *p* < 0.0001 vs. control cells; * *p* < 0.05, *** *p* < 0.001 vs. ATO cells.

The assessment of mRNA expression levels of Nrf2 pathway-related genes after 24 h upon ATO treatment demonstrated the significant upregulation of transcription factor Nrf2 (*** *p* < 0.001) and the downstream inducible enzymes GPX4 (** *p* < 0.01) and HO-1 (**** *p* < 0.0001) compared to the control condition. Recovery with PF administration replaced the expression of Nrf2 pathway-related genes, significantly decreasing Nrf2 (* *p* < 0.05) and HO-1 (*** *p* < 0.001) expression (Figure 7A–C).

## 3. Discussion

Mitochondrial dysfunction plays a pivotal role in statin-induced myopathies and in skeletal muscle disorders due to energy consumers being closely dependent on mitochondrial activity [6]. Despite extensive application of statins, the pathogenesis of statin-induced cardiomyopathy remains unknown. In the present study, our results showed that the exposure of AC16 cells to high concentrations of atorvastatin affected cell viability and induced cell damage. Furthermore, we demonstrated that atorvastatin increased ROS production and induced the loss of cytoskeletal integrity. Previous studies have demonstrated that the administration of atorvastatin in different cellular models increases oxidative stress, reducing GSH levels and increasing lipid peroxidation [13,19]. The massive accumulation of intracellular ROS and lipid peroxidation associated with a decrease in the antioxidant capacity is generally referred to as ferroptosis [20]. In addition to the instability of the plasma membrane and cytoskeleton rearrangement, mitochondrial shrinkage, electron-dense mass formation, and changes in mitochondrial cristae [21] are typical features of ferroptosis. Our microscopy analysis provides convincing evidence that a high concentration of atorvastatin affects the mitochondrial structure and network in human AC16 cardiomyocytes.

Healthy and functioning mitochondria are essential for recovery and regeneration after acute illness. To date, some natural compounds, namely, terpenoids [22], polyphenols [23], flavonoids [24], alkaloids [25], vitamin D [26], etc., are widely known to promote muscle strength, mitochondrial biogenesis, and to reduce hydrogen peroxide production as well as inflammation in skeletal muscles. In this study, we investigated the effect of a natural phytotherapic formulation constituting *Curcuma* and *Boswellia* essential oils, *Harpagophytum procumbens* root, and *Bromelain* on atorvastatin-induced cardiomyopathy in the human AC16 cell line. The results demonstrated that the synergic combination of these extracts was able to improve cardiomyocyte recovery upon atorvastatin injury, reducing ROS production and preserving mitochondria. Specifically, we found that PF increased MMP and induced the expression of SIRT1. Moreover, extensive mitochondrial reshaping associated with an increase in MFN2 mitofusin occurred in AC16 cells supplemented with the phytotherapic extracts. SIRT1 is an important regulator of mitochondrial biogenesis and lipid metabolism [27]. SIRT1 has protective effects on energy metabolism and cardiac stress resistance. It can protect the heart from I/R damage by upregulating antioxidants, downregulating apoptotic molecules and processes, inducing autophagy, and reducing oxidative stress. Overexpression of SIRT1 in cardiomyocytes significantly reduces Nrf2 acetylation exerting cardioprotective effects by regulating oxidative stress. SIRT1 overexpression concomitantly reduces oxidative damage and suppresses PERK/eIF2α/ATF 4 signaling, reducing I/R damage and improving heart function [28]. Nrf2 is a transcription factor considered to be an important regulatory factor in cell antioxidant protection and plays a key role in ferroptosis [29]. Nrf2 is also a prominent player in supporting the structure and function of the stressed mitochondria [30]. Under conditions of oxidative stress, Nrf2 is activated, promoting the transcription of its downstream ferroptosis-related target genes GPx4 and HO-1 [31,32]. In our results, Nrf2 pathway-related genes were significantly upregulated in the recovery after atorvastatin treatment, while PF supplementation replaced this activation, demonstrating the antioxidant activity of PF in mitigating oxidative stress.

This study has mainly two limitations. Firstly, our investigation focused on the antioxidant activity of PF and its protective effect on mitochondria, examining regulation of the Nrf2/HO-1/GPX4 axis. Nevertheless, further investigations are necessary to better characterize the ferroptosis process by analyzing lipid peroxidation. In addition, the employment of animal models is required to validate our experimental findings of the recovery of cardiac damage after PF administration.

## 4. Materials and Methods

### 4.1. Phytotherapic Formulation Composition

The phytotherapic extracts were supplied by GALA Cosmetic srl (Forlì-Cesena, Italy) and kindly provided by Kiron Wellness Lab srl (Bologna, Italy). The detailed product description of the phytotherapic formulation (i.e., name and list of compounds, product and lot numbers, and quality and chemical assessments) is provided in Supplementary Materials (S1_File, S2_File, S3_File, S4_File).

The starting formulation of the phytotherapic extracts was composed of 5% essential oils of *Curcuma* and *Boswellia*, 5% *Harpagophytum procumbens* root extract, and 1% *Bromelain* in medium. This starting formulation was subsequently diluted 1:50, 1:100, and 1:200 to obtain the working solutions tested.

### 4.2. DPPH Test

The antioxidant capacity of the phytotherapic extracts was evaluated in a cell-free system using the 2,2-diphenyl-1-picrylhydrazyl hydrate (DPPH) radical scavenging assay, as previously described [33]. DPPH (100 µM, Sigma-Aldrich, Milan, Italy) and the extract dilutions were prepared in ethanol. The scavenger effect was expressed as % = [(OD 517 nm control) − (OD 517 nm sample/OD 517 nm control)] × 100 and the EC_50_ value was then calculated.

### 4.3. Cell Culture

Human cardiomyocyte AC16 cells were cultured in DMEM/F12 supplemented with 12.5% fetal bovine serum, L-glutamine (100 mM), and 1% antibiotics (penicillin and streptomycin). The cells were incubated in a humidified 5% CO_2_ atmosphere at 37 °C. At 80% confluence, the cells were detached with trypsin–EDTA, washed, and sub-cultivated in new flasks for 1–2 days before the experiments.

### 4.4. In Vitro Model of Atorvastatin-Induced Myopathy and Csell Treatments

Atorvastatin was obtained from Sigma (St. Louis, MO, USA) and was dissolved in dimethyl sulfoxide (DMSO) to make treatment solutions with a final DMSO concentration of 0.1% for all treatments. AC16 cells were treated with increasing concentrations of atorvastatin (1.25, 2.5, 5, 10, and 20 μM) for 24 h. Following treatment, the cells were incubated for an additional 24 h with or without the phytotherapic formulation diluted 1:100 and then analyzed.

### 4.5. Trypan Blue Test

Cell viability was assessed by resuspending the cells in an equivalent volume of 0.4% Trypan Blue solution and then counted using a Burker’s chamber. The number of viable cells in the control condition was set as 100%.

### 4.6. Cell Counting Kit-8 Assay

Cell viability was assessed using the Cell Counting Kit-8 (CCK-8, Sigma-Aldrich, St. Louis, MO, USA, #96992). The reagent, WST-8, is reduced by dehydrogenases in cells to give formazan, a yellow-colored product. Cells were plated on 96-well plates at a density of 7000 cells/well. Following treatment, 10 µL of CCK-8 solution was added to each well. After incubating for 2 h at 37 °C, the absorbance was measured at 450 nm using a microplate photometer (Multiskan FC, Thermo Fisher Scientific, Waltham, MA USA). The cell viability of treated cells was compared to that of control cells.

### 4.7. DCF-DA Assay

Intracellular ROS levels were analyzed with 2’,7’-dichlorofluorescein diacetate (DCFH-DA, Sigma-Aldrich, Milan, Italy), which is a cell-permeable non-fluorescent probe that turns into highly fluorescent 2’,7’-dichlorofluorescein (DCF) upon oxidation [34]. Briefly, cells (7000/well) in black 96-well plates were incubated with DCFH-DA (5 µM) for 30 min at 37 °C. After excess probe removal, the cells were treated with atorvastatin for 1 h. Following treatment, the cells were incubated for an additional 1 h with or without the mixture of natural extracts and then the fluorescence emission was analyzed at ex/em 485/520 nm using a FluoStar Optima (BMG Labtech, Ortenberg, Germany) multiwell plate reader.

### 4.8. Tali Image-Based Cytometric Analysis

Cells were plated on six-well plates at a density of 1 × 10^5^ cells/well. At the end of the treatment, the cells were detached, resuspended in 2.5 µM of Bodipy-TopFluor Cholesterol (Avanti Polar Lipids), and incubated for 30 min at room temperature. The fluorescence was acquired by a Tali™ image-based cytometer (Thermo Fisher) and the data were analyzed with Flow Cytometry Analysis Software (Floreada.io, FCS 3.0).

### 4.9. TMRE Assay

The mitochondrial membrane potential was measured with tetramethylrhodamine, ethyl ester (TMRE, Thermo Fisher Scientific, #T669), a red-orange fluorescent dye readily sequestered by active mitochondria. Cells were plated on black 96-well plates at a density of 7000 cells/well. Following treatment, the cells were incubated with TMRE (100 nM) for 30 min at 37 °C. After excess probe removal, the fluorescence emission was analyzed at ex/em 544/590 nm using a FluoStar Optima (BMG Labtech, Ortenberg, Germany) multiwell plate reader.

### 4.10. Mitochondria Evaluation by MitoTracker Deep Red (MTDR) Staining

AC16 (1 × 10^5^ cells/well) were seeded on glass-bottomed chambers (MatTek Corporation, Ashland, MA, USA). After treatment, the cells were labeled with 200 nM MTDR MitoTracker Deep Red (MTDR; Molecular Probes) at 37 °C for 30 min and observed under a confocal microscope (TCS SP5 II, Leica Microsystem, Wetzlar, Germany). The images were analyzed with NIH-Image J software v1.54f (National Institutes of Health, Bethesda, MD, USA). Form Factor (FF) was measured from the area (Am) and perimeter (Pm) of mitochondria using the formula: FF = Pm^2^/4πAm. Low values of FF indicate a progressive loss of mitochondria morphology and function.

### 4.11. Transmission Electron Microscopy (TEM)

AC16 cells were seeded on glass at a density of 1 × 10^5^ and left to adhere for 24 h. After treatment, the cells were prefixed with 2.5% glutaraldehyde for 1 h at room temperature. The cells were then rinsed with PBS, fixed with 1% OsO4 for 1 h, dehydrated with a series of increasing ethanol concentrations (50–100%), and finally embedded in araldite. Resin blocks were sectioned in semi-thin sections (1–2 µm), stained with 1% toluidine blue, and examined under a light microscope. Ultra-thin sections (70–80 nm) were collected on 400 mesh nickel grids, stained with UranyLess and lead citrate, and viewed under a transmission electron microscope (Philips CM10 at 80 kV). Digital electron micrographs were recorded with a SIS MegaView III camera (Soft Imaging System, Singapore).

### 4.12. Real-Time PCR

Cells were plated on 6-well plates at a density of 1 × 10^5^ cells/well. After treatment, total RNA was extracted using the RNeasy Plus Mini Kit (Qiagen, Milan, Italy), and cDNA was prepared using the PrimeScript RT Master Mix (Takara, Japan). Quantitative RT-PCR was performed using the QuantStudio1 Real-Time PCR system (Thermo Fisher, Milan, Italy). The mRNA expression of target genes [glutathione peroxidase 4 (GPX4), heme oxygenase-1 (HO-1), nuclear factor erythroid 2-related factor 2 (Nrf2), and sirtuin 1 (SIRT1)] was normalized to that of GAPDH, and the relative target gene expression levels were calculated using the 2^ΔΔCT^ method [35]. The primer sequences are reported in Table 1. All procedures were performed according to the manufacturer’s instructions.

### 4.13. Phalloidin Immunofluorescence Analysis

Cells were grown on 35 mm MatTek glass-bottomed dishes (MatTek Corporation; density, 1 × 10^5^ cells/well). After treatment, the cells were washed with PBS, fixed for 15 min in 4% paraformaldehyde, and permeabilized with 0.1% TRITON X-100 for 10 min. The cells were then stained for F-actin by incubation with Phalloidin-FITC solution (P5282, Sigma-Aldrich) (300 nM) for 40 min at RT and labeled with nuclear dye DRAQ5 (Thermo Fisher, 62251) for 10 min at RT. Finally, the cells were observed and analyzed under a Leica TCS SP5 II confocal microscope.

### 4.14. MFN2 Immunofluorescence

Cells were grown on 35 mm MatTek glass-bottomed dishes (MatTek Corporation; density, 1 × 105 cells/well). The cells were fixed for 15 min with 4% (*v*/*v*) paraformaldehyde and then permeabilized with 0.1% Triton X-100 for 15 min at RT. Following treatment, the cells were washed and incubated with blocking solution (PBS containing BSA 2% *w*/*v*) for 60 min. The cells were then incubated overnight at 4 °C with anti-mitofusin-2 (MFN2) (1:50, polyclonal; Cell Signaling Technology, Danvers, MA, USA, #9482) and with conjugated anti-rabbit secondary antibody for 1 h. The cells were labeled with nuclear dye DRAQ5 for 10 min at RT and fluorescent images were captured using a Leica TCS SP5 II confocal microscope (Leica Microsystem, Germany).

### 4.15. Statistical Analyses

Statistical analysis was performed using Prism version 5.00 (GraphPad Software, San Diego, CA, USA). Quantitative data are expressed as the mean ± standard deviation (SD) on the basis of at least three independent experiments. Differences between groups were analyzed using a one-way analysis of variance (One-way ANOVA), followed by Tukey’s post hoc test. The *t*-test was utilized for data comparison between two groups. A value of *p* < 0.05 was indicative of a statistically significant difference.

## 5. Conclusions

Natural products exhibit enormous diversities in their functional groups and have different impacts on the recovery of skeletal muscle damage. However, the synergistic effects of a novel phytotherapic formulation upon statin-induced cardiomyopathy remained unknown. This study demonstrates that the combined supplementation of *Curcuma* and *Boswellia* essential oils, *Harpagophytum procumbens* root, and *Bromelain* counteracts atorvastatin-induced damage in human cardiomyocytes by reducing oxidative stress and promoting the recovery of the mitochondrial network. This work represents a pilot study to launch further investigations of the mechanisms of the cardiotoxicity induced by statins and the protective role of the phytotherapic formulation against cardiomyopathy linked to its scavenging actions and recovery of the mitochondrial network.

## Figures and Tables

**Table 1:** Gene target and primer sequences.

Gene Target	FWD Primer Sequence (5′ → 3′)	REV Primer Sequence (5′ → 3′)
GPX4	CTTCCCGTGTAACCAGTTCG	TCACGCAGATCTTGCTGAAC
HO-1	ATGACACCAAGGACCAGAGC	GTGTAAGGACCCATCGGAGA
Nrf2	AAACCAGTGGATCTGCCAAC	ACGTAGCCGAAGAAACCTCA
SIRT1	CCGGATTTGAAGAATGTTGG	ATCTGCTCCTTTGCCACTCT
GAPDH	AGGTCGGAGTCAACGGAT	TCCTGGAAGATGGTGATG

## Data Availability

Dataset available on request from the authors.

## Supplementary Materials

The following supporting information can be downloaded at: https://www.mdpi.com/article/10.3390/ijms26104917/s1.

## Abbreviations

PF	Phytotherapic formulation (Curcuma and Boswellia essential oils, Harpagophytum procumbens root, and Bromelain)
Nrf2	Nuclear factor erythroid 2-related factor 2
HO-1	Heme oxygenase-1
GPX4	Glutathione peroxidase 4
ETC	Electron transport chain
DPPH	2,2-diphenyl-1-picrylhydrazyl hydrate
DCFH-DA	2’,7’-dichlorofluorescein diacetate
MTDR	MitoTracker Deep Red
TMRE	Tetramethylrhodamine, ethyl ester
MMP	Mitochondrial membrane potential
FF	Form factor
MFN2	Mitofusin-2
SIRT1	Sirtuin 1
ATO	Atorvastatin
TEM	Transmission electron microscopy
