# Supplementary material for: The Activity of Phytotherapic Extracts Combined in a Unique Formulation Alleviates Oxidative Stress and Protects Mitochondria Against Atorvastatin-Induced Cardiomyopathy
Source: Int J Mol Sci. 2025 May 20;26(10):4917. doi: 10.3390/ijms26104917 (PMC12112680; doi:10.3390/ijms26104917)
Supplement: Supplementary file 1 [file ijms-26-04917-s001.zip › ijms-3598179-supplementary/S1_File. ST Harpagophytum procumbens extract.pdf]

TECHNICAL DOCUMENTATION

INCI name: Propylene Glycol, Aqua, Harpagophytum procumbens Root Extract

Product description:

Botanical Name: Harpagophytum procumbens DC.
Plant Part: Root

The extract is obtained by root of Harpagophytum procumbens DC. The plant was cultivated and mechanical harvested. The solvent used for extracton and production is Propylene Glycol.

List of ingredients:

Harpagophytum procumbens Root Extract	10 - 24,9%
Propylene Glycol	> 50%
Aqua	10 - 24,9%
Phenoxyethanol	0.45%
Ethylhexylglycerin	0.055%
Tetrasodium glutamate diacetate	0.047%

Chemical and Quality Assessment:

**Specification**	***Lower Lim. - Upper Lim.***
CAS NUMBER	57-55-6, 7732-18-5, 84988-65-8
EINECS	200-338-0, 231-791-2, 284-853-6
ASPECT	Liquid

GALA srl SB - Via Antonio Cicognani, 11/N - 47100 FORLI' (FC) Italy - Tel. +39.0543.781.838 - Fax +39.0543.782.598
P.IVA 03429850401 - E-Mail: info@galacosmetici.com

ODOUR	Characteristic
COLOUR	Dark brown
DENSITY (20°C)	1,045 - 1,060 g/ml
DRY RESIDUE (180°C, 30')	>= 1,00%
WATWER SOLUBILITY	Soluble
pH	5,0 - 7,0
PRESERVATIVES	Phenoxyethanol 0.45 %,<br>Ethylhexylglycerin 0.05 %,<br>Tetrasodium glutamate diacetate 0.047 %
TOTAL AEROBIC COUNT	<=100 UFC/g
YEAST AND MOULD	<= 10 UFC/g
S. AUREUS	Absent in 1 g
P. AEROUGINOSA	Absent in 1 g
CANDIDA ALBICANS	Absent in 1 g
HEAVY METALS	<=20,0 ppm
Pb, Cd, Cr, Hg, As, Ni	Respectively < 1 ppm

Lot Number:

V0308201
