# Supplementary material for: The Activity of Phytotherapic Extracts Combined in a Unique Formulation Alleviates Oxidative Stress and Protects Mitochondria Against Atorvastatin-Induced Cardiomyopathy
Source: Int J Mol Sci. 2025 May 20;26(10):4917. doi: 10.3390/ijms26104917 (PMC12112680; doi:10.3390/ijms26104917)
Supplement: Supplementary file 1 [file ijms-26-04917-s001.zip › ijms-3598179-supplementary/S2_File. ST Bromelain.pdf]

TECHNICAL DOCUMENTATION

INCI Name: BROMELAIN, MALTODEXTRIN

Product description:

Botanical Name: Ananas comosus
Plant Part: Pineapple stipites
Complete scientific name: *Bromeliaceae, Ananas, Ananas comosus* (L.) Merr.

The extract is obtained by Pineapple stipites from India. The plant was cultivated and mechanical harvested. The solvent used for extracton and production is water.

List of ingredients:

**Ingredients**	**Composition**
Bromelain	80 – 85%
Maltodextrin	15 – 20%

Chemical and Quality Assessment:

**Specification**	***Lower Lim. - Upper Lim.***
CAS NUMBER	9001-00-7/9050-36-6
EINECS	232-572-4/232-940-4
ASPECT	Free-flowing, hygroscopic powder
ODOUR	Characteristic
COLOUR	White to off-white powder
PARTICLE SIZE	100% passes throught 40 mesh
LOSS ON DRYING	<= 8,00 %
pH	5,0 - 8,0
IDENTIFIABLE ACTIVITY	Positive for proteolytic activity

GALA srl SB - Via Antonio Cicognani, 11/N - 47100 FORLI' (FC) Italy - Tel. +39.0543.781.838 - Fax +39.0543.782.598
P.IVA 03429850401 - E-Mail: info@galacosmetici.com

PROTEOLITIC ACTIVITY	>= 2.500 GDU/g
PRESERVATIVES	//
TOTAL AEROBIC COUNT	<=10.000 CFU/g
YEAST AND MOULD	<= 100 CFU/g
E. COLI	Absent/25g
SALMONELLA	Absent/10g
LEAD	<=3 ppm
MERCURY	<=1 ppm
CADMIUM	<=1 ppm
ARSENIC	<=1 ppm
HEAVY METALS	<=10 ppm

Lot Number:

V0977202
