# Supplementary material for: The Activity of Phytotherapic Extracts Combined in a Unique Formulation Alleviates Oxidative Stress and Protects Mitochondria Against Atorvastatin-Induced Cardiomyopathy
Source: Int J Mol Sci. 2025 May 20;26(10):4917. doi: 10.3390/ijms26104917 (PMC12112680; doi:10.3390/ijms26104917)
Supplement: Supplementary file 1 [file ijms-26-04917-s001.zip › ijms-3598179-supplementary/S3_File. ST Boswellia carterii.pdf]

TECHNICAL DOCUMENTATION

INCI Name: BOSWELLIA CARTERII GUM OIL

Product description:

Botanical Name: Boswellia carterii

Plant Part: Gum

Origin: Somalia

Pure and natural essential oil obtained by steam distillation of gum of Boswellia carterii from Somalia.

List of ingredients:

ANNEX III - Substances restricted according to their use, listed in Annex III of the Cosmetic Regulations:

Substance:	CAS:	EC:	%
PINENE	80-56-8	201-291-9	14.6
D-LIMONENE	5989-27-5	227-813-5	13.75
BETA-CARYOPHYLLENE	87-44-5	201-746-1	1.85
DELTA-3-CARENE	13466-78-9	236-719-3	0.6
GAMMA-TERPINENE	99-85-4	202-794-6	0.5
LINALOOL	78-70-6	201-134-4	0.325
CARVONE	6485-40-1 | 99-49-0 | 2244-16-8	229-352-5 | 218-827-2 | 202-759-5	0.309
L-BETA-PINENE	18172-67-3	242-060-2	0.3
TERPINOLENE	586-62-9	209-578-0	0.15
TOLUENE	108-88-3	203-625-9	0.05
TERPINEOL	8000-41-7	232-268-1	0.016
BENZALDEHYDE	100-52-7	202-860-4	0.015
FURFURAL	98-01-1	202-627-7	0.005
BENZYL BENZOATE	120-51-4	204-402-9	0.005
METHYL EUGENOL	93-15-2	202-223-0	0.001

Chemical and Quality Assessment:

Specification	*Lower Lim. - Upper Lim.*
ASPECT	Viscous liquid
ODOUR	Compliant
COLOUR	From Yellow To Amber
DENSITY (20°C)	0.956 (+/-0,01)
REFRACTIVE INDEX	1.503 (+/-0,01)

Lot Number:

ORA23002258
