# Supplementary material for: The Activity of Phytotherapic Extracts Combined in a Unique Formulation Alleviates Oxidative Stress and Protects Mitochondria Against Atorvastatin-Induced Cardiomyopathy
Source: Int J Mol Sci. 2025 May 20;26(10):4917. doi: 10.3390/ijms26104917 (PMC12112680; doi:10.3390/ijms26104917)
Supplement: Supplementary file 1 [file ijms-26-04917-s001.zip › ijms-3598179-supplementary/S4_File. ST Curcuma longa.pdf]

TECHNICAL DOCUMENTATION

INCI Name: CURCUMA LONGA ROOT OIL

Product description:

Botanical Name: Curcuma longa
Plant Part: Rhizome
Origin: India

Pure and natural essential oil obtained by steam distillation of rhizoma of Curcuma longa from India.

List of ingredients:

ANNEX III - Substances restricted according to their use, listed in Annex III of the Cosmetic Regulations:

Substance:	CAS	EC	%
TUMERIC	8024-37-1	283-882-1	25-50
BETA-CARYOPHYLLENE	87-44-5	201-746-1	1 -10
CINEOLE	470-82-6	207-431-5	1 -2,5
LIMONENE	5989-54-8	227-815-6	1 -2,5
CYMENE	99-87-6	202-796-7	0,1 -2,5
BISABOLENE	495-61-4		0,1 -1

Chemical and Quality Assessment:

**Specification**	***Lower Lim. - Upper Lim.***
ASPECT	Liquid
ODOUR	Compliant
COLOUR	From Pale Yellow To Brownish Yellow
DENSITY (20°C)	0.92 (+/-0,01)
REFRACTIVE INDEX	1.505 (+/-0,01)

GALA srl SB - Via Antonio Cicognani, 11/N - 47100 FORLI' (FC) Italy - Tel. +39.0543.781.838 - Fax +39.0543.782.598
P.IVA 03429850401 - E-Mail: info@galacosmetici.com

Lot Number: OO091122
